# The demographic features, clinical outcomes, prognosis and treatment options for patients with sarcomatoid carcinoma of the urinary bladder: a single centre experience

**DOI:** 10.1590/S1677-5538.IBJU.2016.0347

**Published:** 2018

**Authors:** Simon Paul Robinson, Assad Farooq, Marc Laniado, Hanif Motiwala

**Affiliations:** 1 Frimley Health Foundation Trust - Urologia, Wexham Street, Slough, United Kingdom, UK; 2 Heatherwood and Wexham Park Hospitals NHS Trust, Wexham Park Hospital Wexham Slough, Slough, United Kingdom, UK; 3 Department of Urology, Heatherwood and Wexham Park Hospitals NHS Trust - Slough, Berkshire, United Kingdom, UK

**Keywords:** Urinary Bladder, Sarcoma, Carcinoma

## Abstract

**Introduction:**

Carcinosarcoma of the bladder is a very rare neoplasm. The pathogenesis of carcinosarcomas is not clearly understood and remains a subject of debate. Whilst there is some research conceptualizing the histopathological findings of bladder carcinosarcomas, the demographic features, clinical outcomes, prognosis and treatment options remain unclear.

**Materials and Methods:**

We analyzed 12 consecutive cases of patients with sarcoma-toid bladder cancer who were treated surgically at a single Urology Department be-tween 1999 and 2015. Radiology, pathology and surgical reports were reviewed to determine the pathological staging at the time of cystectomy. These were directly compared with 230 patients having cystectomies for urothelial cell carcinoma. The sarcomatoid patients, were compared to patients with urothelial cell cancers. The other histological sub types, squamous cell (17), neuroendocrine (9), metastatic (7), mixed (4), adenocarcinoma (3), were not included.

**Results and conclusion:**

Carcinosarcoma of the urinary bladder is often described in the literature as a highly malignant neoplasm that is rapidly lethal. We found that the sarcoma does not offer a worse prognosis than conventional high-grade urothelial car-cinoma. There is no significant difference in grade, stage, positive surgical margin rate, nodal involvement, associated prostate cancer or incidence rates of progression, all cause or disease specific mortality. There was a barely significant difference in carcinoma in-situ. However, carcinosarcomas are three times the volume of urothelial cell tumors which may contribute to its reputation as an aggressive tumour (44cc v 14cc). Sarcomatous elements do not appear, from our small study, to bestow a worse prognosis.

## INTRODUCTION

The World Health Organization defines sarcomatoid carcinoma, also known as carcinosarcoma, as a biphasic tumour consisting of malignant epithelial and mesenchymal cells (1). Carcinosarcoma of the bladder is a very rare neoplasm with extremely low number of cases reported in the literature from as early as 1972.

The pathogenesis of bladder carcinosarcomas is not clearly understood and remains a subject of debate. A comparative genomic hybridization study undertaken by Völker et al. (2) suggests that the epithelial and mesenchymal components of cases revealed important similarities. Remnants of epithelial cell surface markers and ultrastructural features were shown to be present in mesenchymal and sarcomatoid components. They hypothesize that carcinosarcomas are the end products of different pathways of differentiation of upstream totipotential neoplastic cells. However, in instances where the different components share no histochemical similarities, Gorstein et al. (3) propose that carcinosarcomas result from so-called collision tumors in which epithelial and mesenchymal components arise separately. It should be noted that the tumour components showed clonal identity which would support a monoclonal origin (4-6).

Earlier research in this field suggests that the microscopic morphology of carcinosarcomas comprises a variable combination of sarcomatous and carcinomatous constituents. In the vast majority of reported cases, the epithelial component is essentially high-grade urothelial cell carcinoma, while the sarcomatous constituents can consist of chondrosarcoma, osteosarcoma, leiomyosarcoma, histiocytoma, fibro sarcoma or rhabdomyosarcoma (7-9).

Whilst there is some research conceptualizing the histopathological findings of bladder carcinosarcomas, the demographic features, clinical outcomes, prognosis and treatment options remain unclear.

The objective of this study was to analyze 12 consecutive cases of patients with muscle-invasive or metastatic sarcomatoid bladder cancer who were treated at a single Urology Department between 1999 and 2016. This retrospective analysis was carried out to gain more understanding regarding the clinical behavior, treatment and outcome of this aggressive disease. This is the first study which compares the outcomes of carcinosarcomas with urothelial cell carcinoma (TCC), which can help to put the behavior of carcinosarcoma patients in clinical perspective.

## MATERIALS AND METHODS

### Study population

This is a retrospective case series in which we reviewed the medical records of all patients with sarcomatoid bladder carcinoma treated with radical cystectomy at our cancer centre between 1999 and 2015.

### Case selection

We searched our hospital patient database and selected patients with established sarcomatoid disease and for whom the pathology report revealed any sarcomatoid component in their tumor. Although cystectomy patients had their prior TURBT analyzed, not all TURBT specimens with sarcomatous elements were searched for. Patient medical records were carefully reviewed to assess the demographic characteristics, clinical stage and outcome. The patients in our study were followed up in clinic annually. Our primary end-point was patient mortality and we calculated our survival data by comparing the disease course for each patient. These were processed into Kaplan-Meier curves of survival. We followed up our patients who were alive during the conduct of the study in our clinic. The survival data was based on analysis of our hospital medical records which recorded morbidity, mortality and each detail of each hospital admission or episode.

### Tumour characteristics

Radiology, pathology and surgical reports were reviewed to determine the pathological staging at the time of cystectomy using the 2009 TNM classification for genitourinary tumors.

Chemotherapy regimens, radiotherapy doses, and surgical modality were also recorded. In order to compare the clinical characteristics and outcomes of patients with sarcomatoid components with those who did not have these components, patients who had sarcomatoid components were compared to patients in our bladder cancer database who had no sarcomatoid elements present.

The histopathological slides were analyzed by the Pathology Department. The site of the tumor was not recorded and any correlation/relation between TURBT and cystectomy was not possible.

### Statistical tests

GraphPad (10) and MedCalc version 13 (11) Fishers exact test, Mann Whitney U tests, t-test, incidence rates, log rank and Kaplan-Meier curves (Medcalc) were used.

## RESULTS

### Patient characteristics

The mean age for patients with TCC was 67 as compared to 70 for patients with sarcomatoid-carcinoma (U-test=0.37). There were 185 males and 45 female patients in the TCC cohort as compared to 9 males and 3 females in the sarcomatoid cohort (Fisher=0.72).

### Histological results

We compared different histological characteristics of the sarcomatoid (n=12) cohort of patients with those with TCC (n=230). Although the number of sarcomatoid cases was small, this provided an interesting comparison to our understanding of the natural development of sarcomatoid tumors. Our patients showed a variety of subtypes with both epithelial and sarcomatous elements (Table-1). The epithelial component was urothelial cell in 8 cases, squamous in 3 cases, and unidentified epithelium in 1 case. There was no significant difference in the grade or stage of tumor between the patients undergoing cystectomy for urothelial cell or sarcoma, although the confidence intervals are wide because of the small number of sarcomatoid cases (Table-2). Patients with sarcoma had much larger tumors (43cc) as compared with patients with urothelial cell (14cc). There was a tendency to more CIS with urothelial cell carcinoma with nearly half the cases featuring this. There was no difference in the rate of nodal metastasis, or in the rate of extracapsular extension of the metastasis. There was a significant difference in the nodal density with the one sarcomatoid patient with nodal deposits having 12/22 nodes involved compared to 128/2299 (0.05%) with TCC. This is almost certainly a significant statistical finding rather than a genuine clinical finding (a type 1 error). Neither was there a significant difference in the positive margin rate, in the rate of additional treatment or of prostate cancer (Table-2).

**Table 1:** Patient characteristics and histopathological information.

Case	Age	Sex	T stage	Tumor volume	Histology sarcomatoid	Histology epithelial	Comments
1	57	Male	2	24	Carcinosarcoma; Chondroid metaplasia; Neuroendocrine differentiation	High grade urothelial cell carcinoma	Squamous metaplasia
2	77	Male	1	30	Carcinosarcoma; Spindle cell, malignant cartilage; Smooth muscle antigen positive	Low grade urothelial cell carcinoma	
3	66	Male	3	18	Sarcomatoid; Vimentin positive	High grade urothelial cell; Epithelial marker antigen positive	Squamous metaplasia
4	81	Female	2	38	Carcinosarcoma; Vimentin positive	Squamous cell carcinoma; Squamous carcinoma in situ	Squamous metaplasia
5	77	Male	3	10.5	Carcinosarcoma with angiosarcomatous differentiation; Anaplastic spindle cell	Squamous cell carcinoma	
6	77	Male	2	153	Sarcomatoid carcinoma; Spindle and dendritic cell; Vimentin positive	High grade urothelial cell	
7	74	Female	2	20	Primary sarcoma/sarcomatoid carcinoma; Spindle/Rhabdoid and polygonal cells occasional stellate cells, focal myxoid stroma; Heavy eosinophilic infiltrate with scattered plasma cells; CD68 positive; Desmin and Alk-1 (CD246) Caldesmon negative; Smooth muscle actin/myosin positive Eosinophilic Charcot-leyden crystals; Widespread necrosis with calcification; Frequent mitotic figures	Epithelial marker antigen cytokeratin positive; 2^nd^ opinion Oxford; 3^rd^ opinion Royal Marsden Hospital	2^nd^ opinion John Radcliffe; Oxford; 3^rd^ opinion Royal Marsden; Confirmation
8	75	Male	2	37	Neuroendocrine with sarcomatoid differentiation	High grade urothelial cell	
9	69	Male	2	7	Sarcomatoid	High grade urothelial cell	
10	62	Male	3	32	Solid sarcomatoid carcinoma; Spindle cells within myxoid background; necrosis	Poorly differentiated Squamous and urothelial carcinoma	Squamous metaplasia
11	79	Male	3	80	Sarcomatoid urothelial carcinoma, leiomyosarcoma; Spindle cells, mitotic figures abundant; Necrosis, myxoid regions; SMA, Vimentin, CD10 positive; Epithelial marker negative; Desmin, Caldesmon negative	Epithelioid regions; Pan CK, 34 beta E12, p63, vimentin, EMA positive	Squamous metaplasia; 2^nd^ opinion John Radcliffe; 3^rd^ opinion Nuffield orthopedic; Oxford; Favoring sarcomatoid urothelial carcinoma over leiomyosarcoma
12	46	female	3	102	Non-specific sarcomatoid differentiation	High grade urothelial carcinoma; necrosis	

**Table 2:** Demographic and clinicopathological characteristics of urothelial and sarcomatoid bladder tumurs.

		Sarcomatoid	Urothelial cell	P value
Male/female		9/3	185/45	0.726
Age years		65.3	67.67	0.37
Grade high		10	213	0.269
	Intermediate	2	17	
Tumour volume cc		44	14	0.0015
T stage localized		7	140	0.771
Locally advanced		5	90	
Carcinoma in-situ		2/12	105/230	0.0428
Nodal involvement		1/12	51/230	0.3096
Nodal density		12/22 = 0.545	128/2299 = 0.055	0.0001
Extracapsular extension		1/12 = 0.083	22/128 = 0.44	0.69
Prostate cancer		5/7 = 0.71	83/185 = 0.44	0.14
complications		2/12= 0.17	59/230 = 0.25	0.044
Additional treatment (chemo or radiotherapy)		3/12 = 0.25	44/230 = 0.19	0.29
Positive surgical margins		0/12	21/230	1.0

**Figure 1:**
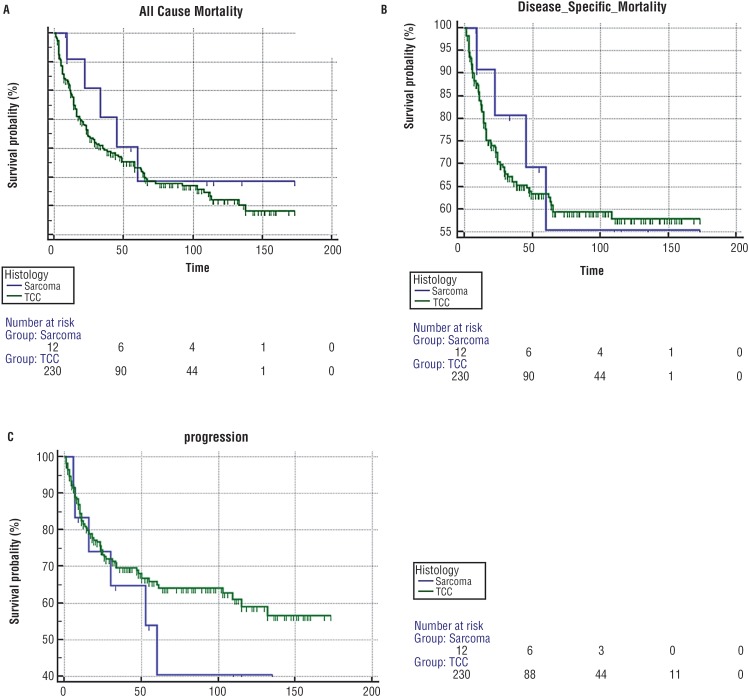
A) Kaplan-Meier curve demonstrating all cause mortality. 1B) Kaplan-Meier curve demonstrating disease specific mortality. 1C) Kaplan-Meier curve demonstrating time to progression.

There was no significant difference for rates of progression, overall or disease specific mortality between the two cohorts (Table-3, Figure-1).

**Table 3:** Survival rates among urothelial and sarcomatoid bladder tumors.

	Urothelial	Sarcomatoid	Log rank significance test
All causes of mortality (mean survival in months)	89 (CI 79-100)	102 (CI 57.756 to 146.385)	P=0.52
Disease specific survival (mean survival in months)	110 (CI 99-120)	104 (CI 61-149)	P=0.61
Progression free survival (mean survival in months)	106 (CI 95-117)	74 (CI 40.8 – 106.8)	P=0.61

## DISCUSSION

Carcinosarcoma of the urinary bladder is often described in the literature as a highly malignant neoplasm which is rapidly lethal. They may be collision tumours of urothelial and sarcomas, poorly differentiated urothelial carcinoma with osteocartilaginous stromal metaplasia, or undifferentiated sarcomatoid spindle cell urothelial carcinoma. In true carcinosarcoma, epithelial and mesenchymal components are randomly mixed. Immunohistochemically, the epithelium is characterized by cytoplasmic cytokeratin expression and the sarcomatous element by vimentin, desmin and muscle specific actin (12). There can be crossover of markers. Epithelial antigens, pankeratin, EMA, can be found in sarcomatous tissue. Similarly, cell adhesion molecules, E cadherin, CD44, CD44v6 are found to varying degrees in both (13).

We have found that the sarcoma does not offer a worse prognosis than conventional high-grade urothelial carcinoma. Our findings do not corroborate with an earlier series of cases published in 1998 by Lopez-Beltran et al. who reported that bladder sarcomas are aggressive malignancies with a mean survival of 10 to 17 months (8). A recent case series by Wang et al. in 2010 with a larger cohort (n=221) also reiterated the highly aggressive nature of the disease (14). They also built on the earlier case series by emphasizing the need for early detection, including the identification of risk factors to improve the overall clinical outcomes.

Regarding the rarity of these tumours, Helpap (12) found 0.5% of bladder tumours were nonepithelial similar to other series. However, our rate is ten times this at 4.5%; these variations will no doubt occur with small series.

Currently the field of uro-oncology for carcinosarcoma of the urinary bladder is bereft of randomized controlled trials, given the rarity of this disease. As a result, there is no standard treatment of choice. Indeed, there is debate about whether surgical treatment alone or a multi-modality approach would be most efficacious. In a third case series (n=14) by Wang et al., aggressive multi-modal treatment with the sequential use of chemoradiotherapy after surgical resection in 3 out of 14 patients led to a complete response and markedly improved survival (4). Four of our surgical patients had multimodal treatment. Two had adjuvant gemcitabine and cisplatin and they are still alive at 118 and 8 months respectively. One patient had neo-adjuvant radiotherapy (20 Gray) but died after 45 months. Another had neo-adjuvant MVAC and died 9 months post surgery.

Despite the initial appearance of worse survival outcomes for carcinosarcoma patients, we have not found any significant difference between the two cohorts Figure 1a: all cause mortality, Figure 1b for disease specific mortality and Figure 1c for progression free survival.

Sarcomatous tumors were three times the volume of urothelial cell tumors in this report (mean 43cc v 14cc), which may contribute to its reputation as an aggressive tumour. However, there was less CIS associated, which may contribute to the better outcome for the patients reported here. On cox regression analysis, histology does not, regardless of type, bestow a worse prognosis. The most important prognostic factors are stage related. Sarcomatoid elements should not darken the attitude of physicians, or patients, and allow them to better assess the risks and potential benefits of treatment. Sarcomatoid tumours, like high grade TCC is an aggressive tumour, but no more so than its urothelial counterpart. We hope that our study adds to the very small pool of studies done sporadically over the last 3 decades and stimulates further debate on this subject. Further advances in the molecular biology of this disease may lead to development of targeted treatment strategies for this very rare but dangerous disease.

## LIMITATIONS

Limitations of our study include the retrospective nature of its design and the small number of patients, which is unavoidable due to the rarity of the disease. We do plan to compare all TURBT specimens showing sarcomatoid components to illustrate the range of treatments and outcomes compared to those receiving cystectomy. This, however, is not too dissimilar to the study sizes of previously published case series (Lahoti et al. n=5, Wang et al. n=14) (7, 15).

## CONCLUSIONS

From this study, it does not appear that sarcomatoid tumors of the bladder bestow a worse prognosis.
